# Relapse in childhood B-cell precursor acute lymphoblastic leukemia: insights from transcriptomic signatures of diagnostic bone marrow

**DOI:** 10.3389/fonc.2026.1778311

**Published:** 2026-04-15

**Authors:** Lixian Oh, Mohamad Shafiq Azanan, Hany Ariffin

**Affiliations:** Department of Paediatrics, Faculty of Medicine, Universiti Malaya, Kuala Lumpur, Malaysia

**Keywords:** B-cell precursor acute lymphoblastic leukemia, cell death, drug resistance, immune modulation, predictive biomarkers, relapse, stemness, transcriptome

## Abstract

Survival rates exceeding 85% are now achieved for most children diagnosed with B-cell precursor acute lymphoblastic leukemia (BCP-ALL), the most common subtype of childhood ALL. Advances in the understanding of disease biology, the identification of robust prognostic factors, and the implementation of risk-adapted treatment protocols have enabled increasingly personalized therapeutic approaches. The integration of RNA sequencing into routine diagnostics has further refined subtype classification and facilitated the detection of cryptic rearrangements and pathogenic variants. Despite these improvements, a subset of patients still experience relapse—even those with favorable clinical and cytogenetic features as well as early clearance of residual disease. This overview underscores persistent gaps in current risk-stratification strategies; an important consideration, particularly for children assigned to low-risk groups who consequently receive reduced-intensity therapy. In this review, we synthesize current evidence on the diagnostic transcriptomic profiles in BCP-ALL that are associated with relapse, framing the discussion around five interconnected biological domains, namely cell death dysregulation and stress-adaptive survival; immune modulation and metabolic adaptation; developmental dysregulation and stemness; drug resistance; and post-transcriptional regulation. We focus specifically on transcriptomic signatures detectable at initial diagnosis, which reflect intrinsic leukemic cell states prior to therapeutic exposure, that may provide early indicators of relapse susceptibility. With the increasing availability of RNA-sequencing data across treatment centers, new opportunities are emerging to analyze these datasets beyond conventional subtype assignment. Comprehensive interrogation of transcriptomic profiles may help identify relapse-associated signatures that complement established clinical and genomic markers. As contemporary protocols continue to explore treatment de-intensification to minimize long-term toxicities, it is essential to ensure that patients classified as “low-risk” are accurately identified and do not harbor occult molecular features that predispose to relapse. This review provides a narrative overview of the transcriptomic determinants of relapse in BCP-ALL at diagnosis and highlights key knowledge gaps. By delineating the molecular pathways that may contribute to relapse, we aim to improve relapse prediction and provide a framework for future development of precise, personalized therapeutic strategies.

## 1 Background

B-cell precursor acute lymphoblastic leukemia (BCP-ALL) is the most common cancer in childhood, accounting for 80-85% of childhood ALL cases (1). In 2021, the prevalence rate was 25.66 per 100,000 children aged 0–14 years (2). The standard therapeutic approach for most children with BCP-ALL is multi-agent chemotherapy administered in phases. While different pediatric oncology groups may investigate distinct research questions and therapeutic refinements, the backbone of modern childhood ALL therapy remains largely consistent across institutions, comprising a structured treatment course over approximately two years, with each phase incorporating specific combinations of chemotherapeutic agents. However, immunotherapy, targeted agents, and bone marrow transplantation are also used in certain cases.

BCP-ALL is a heterogeneous disease; accordingly, therapy is individualized based on a combination of clinical, genetic, and molecular risk factors. At diagnosis, leukemic cells (lymphoblasts) are characterized by their morphological, immunophenotypic and cytogenetic features obtained from bone marrow aspirate, allowing categorization into broad disease subtypes.

Treatment intensity is adjusted according to patient- and leukemia-related predictors of outcomes, including age, white blood cell (WBC) count, central nervous system (CNS) involvement, immunophenotypes, cytogenetic subtype, specific chromosomal or molecular aberrations, such as *IKZF1* alterations (3, 4) and importantly, response to therapy as measured by minimal residual disease (MRD) i.e. presence of sub-microscopic amounts of leukemia cells at the end of designated phases of the treatment regimen (5). MRD, assessed via either flow cytometry or PCR-based assays, provides a highly sensitive measurement of treatment effectiveness and remains one of the strongest predictors of outcome in childhood ALL. Collectively, these parameters form the foundation of contemporary risk-adapted treatment protocols.

These approaches have translated into substantial improvements in disease outcome, with the overall survival for childhood BCP-ALL now reaching 85-90% in many regions (6–10). Despite these successes, relapses were still reported in up to 15% of patients. Importantly, relapse is not confined to patients classified as high-risk at diagnosis; a subset of patients deemed low-risk based on conventional clinical, cytogenetic, and MRD criteria still experience disease recurrence (11). This discordance suggests that knowledge gaps still exist in our understanding of leukemia biology, and clinically relevant biological determinants/markers of relapse risk are not fully captured by existing stratification approaches.

Genomic and transcriptomic profiling has emerged as a powerful approach to interrogate leukemia biology beyond classic genetic lesions. Beyond refining disease subgroups, interrogation of the blast genome has helped uncover hitherto unknown molecular drivers of leukemogenesis and thereby improve more precise risk stratification and treatment tailoring. High-throughput platforms such as RNA sequencing (RNA-seq) and microarrays enable comprehensive profiling of gene expression patterns, detection of alternative splicing, identification of cryptic or novel chromosomal rearrangements, and regulatory network activity that reflect functional cell states already present at diagnosis. Accumulating evidence indicates that such transcriptional signatures can anticipate relapse risk, independently of, or in addition to, established clinical and genetic predictors, providing both prognostic value and mechanistic insight into treatment resistance.

In this review, we synthesize evidence from transcriptomic studies that leverage diagnostic bone marrow samples to identify molecular features associated with subsequent relapse. The broadly similar therapeutic backbone used across modern treatment protocols provides a shared biological context for interpreting transcriptomic predictors of relapse across independent cohorts. We focus specifically on transcriptomic profiles obtained at initial diagnosis, as these signatures reflect intrinsic leukemia cell states present before chemotherapy exposure. While transcriptomic alterations observed at relapse provide important insights into clonal evolution and acquired resistance, such changes often represent secondary adaptations arising under therapeutic pressure and thus fall outside the primary scope of this review. Instead, emphasis is placed on identifying early transcriptomic signatures at diagnosis that may serve as potential early predictors of subsequent relapse.

Most large-scale transcriptomic studies investigating diagnostic predictors of relapse have been conducted in pediatric BCP-ALL, for which large, well-annotated clinical datasets are available. Accordingly, the transcriptomic signatures discussed in this review predominantly reflect BCP-ALL biology. Where studies included heterogeneous ALL populations without lineage-specific analyses, this fact is explicitly noted.

**Figure 1:**
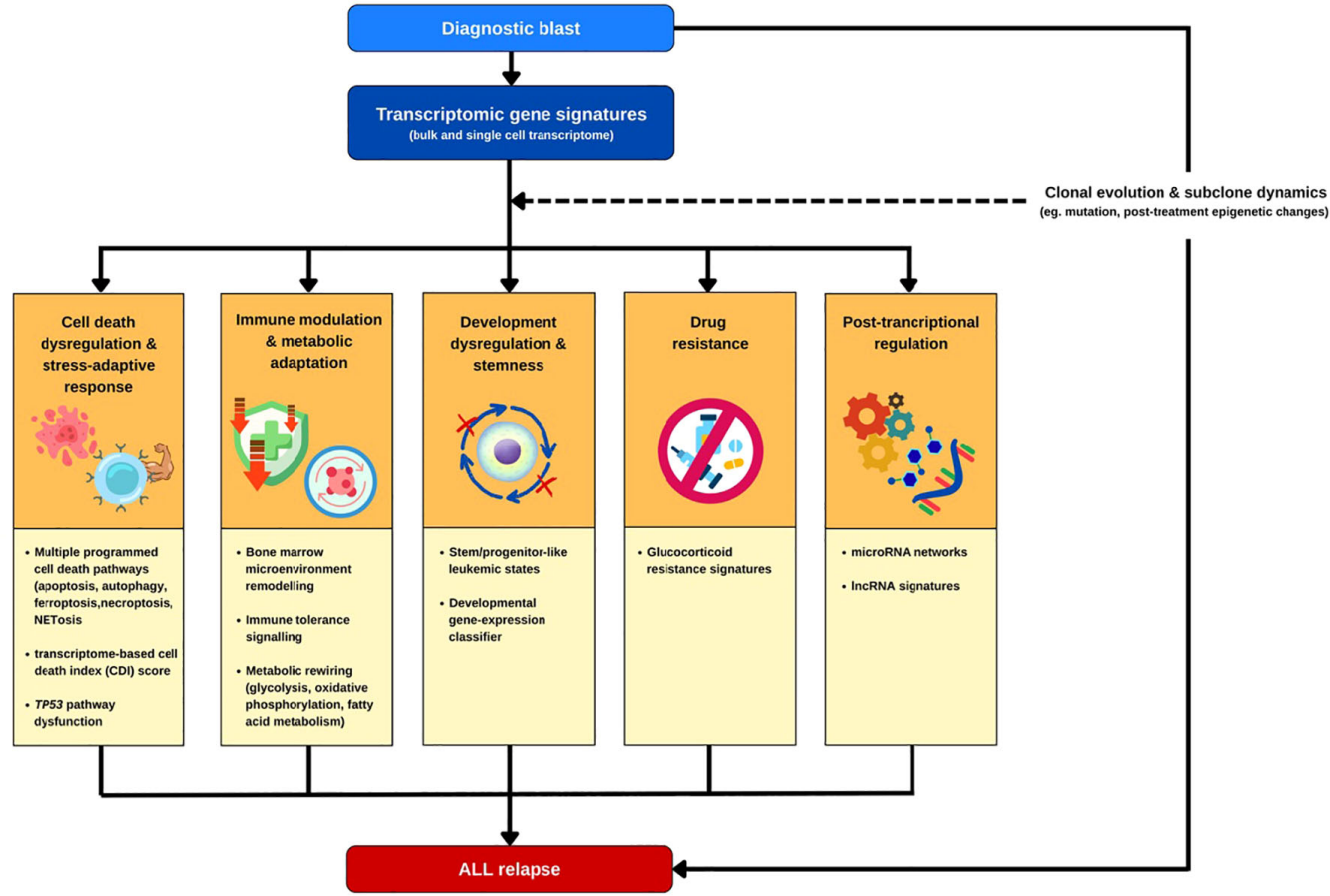
Transcriptomic gene signatures at diagnosis reveal biological processes associated with relapse risk in pediatric B-cell precursor acute lymphoblastic leukemia (BCP-ALL). Diagnostic transcriptomic signatures, including protein-coding genes, miRNAs, and lncRNAs, capture key biological domains that drive leukemic persistence and relapse: cell death dysregulation and stress-adaptive survival; immune modulation and metabolic adaptation; developmental dysregulation and stemness; and drug resistance. Representative signatures and key signaling pathways are indicated within each domain. Clonal evolution and subclone dynamics are included as an additional component, distinct from the diagnostic transcriptional signatures, reflecting the evolving clonal architecture that may influence relapse risk. Integrating these transcriptomic signatures provides a systems-level framework for understanding and predicting relapse risk.

To provide a structured framework, we organized the relapse-associated transcriptome findings into five biological domains, including 1) cell death dysregulation and stress-adaptive survival, 2) immune modulation and metabolic adaptation, 3) dysregulated development and stemness, 4) drug resistance and 5) post-transcriptional regulation (**Figure 1**), and discuss how transcriptional signatures across these domains enhance early relapse prediction and provide insights into biological processes associated with therapeutic failure. **Table 1** summarizes representative diagnostic transcriptomic signatures linked to relapse risk, organized according to these domains, which are discussed in detail in the sections below.

**Table 1:** Key transcriptomic signatures and associated biological domains linked to relapse risk in pediatric ALL.

Domain	Transcriptomic features	Mechanistic insight/pathways	Clinical/predictive relevance	Reference
Cell Death Dysregulation and Stress-Adaptive Survival Mechanisms	*BIK, BCL2L2, WWOX*	Apoptosis	Contributes to cell death index (CDI) score; predictive of relapse	(12)
	*TSPO, PIP4K2C*	Autophagy, mitochondrial function, metabolism	Contributes to CDI score; predictive of relapse	(12)
	*MLKL, STAT2*	Necroptosis, immune dysregulation,	Contributes to CDI score; predictive of relapse	(12)
	*TP53*	Apoptosis, cell cycle, DNA repair	High expression predictive of relapse	(20)
Immune modulation & metabolic adaptation	*SIRT3, NADSYN1*	NAD+ metabolism, mitochondrial function	Correlates with reduced naive B-cell at relapse	(22)
	*PARP6*	NAD+ metabolism	Correlates with expansion of neutrophils at relapse	(22)
	*IF144L*	Interferon-stimulated immune response	Low expression is associated with early relapse risk	(30)
	*TIMD4*	Immune tolerance and impaired antigen presentation	High expression predictive of early relapse	(23)
	Interferon and inflammatory pathways	Immune activation	Correlates negatively with CDI scores which predicts relapse	(12)
	Metabolic signatures (glycolysis, fatty acid metabolism, oxidative phosphorylation, MTORC1, MYC signaling)	Metabolic reprogramming	Correlates positively with CDI scores which predicts relapse,	(12)
	59-gene relapse-free survival biomarker	Chemokine/cytokine signaling, cell-cycle regulation, intracellular trafficking, lineage markers	Combined expression model stratifies 2 risk groups, high risk shows poorer relapse-free survival	(26)
	*CD19*	B cell lineage marker, altered B-cell receptor signaling	High expression predictive of CNS relapse	(27)
	*PPARG*	Lipid metabolism, mitochondrial function,	Low expression predictive of CNS relapse	(27)
	*GNG12*	G-protein signaling, endothelial junction integrity	High expression predictive of CNS relapse	(27)
Developmental dysregulation & stemness	38-gene relapse-free survival expression classifier	Stemness-associated transcriptional programme, B-cell development and differentiation, cell signaling regulation	Combined with flow MRD, expression model stratifies 3 risk groups high risk shows poorer relapse-free survival	(29)
	*HOXA7*	Cellular Differentiation blockade, cell-cycle regulation	High expression predictive of early relapse	(30)
	*S100A10, S100A11*	Calcium-binding, cytoskeletal dynamics, cell-cycle progression, stress adaptation	High expression predictive of early relapse, chemoresistance	(30)
Drug resistance	*B4GALT5, CDK6, PDZ8, RAB7A*	Glucocorticoid resistance	Subset of 59-gene relapse free survival signature, associated with prednisolone response	(26)
	NLRP3-CASP1 inflammasome	Glucocorticoid resistance	Increased CASP1 and NLRP3 at diagnosis associated with glucocorticoid resistance and poorer disease-free survival	(37)
	Prednisone-dependent expression signature,	Glucocorticoid resistance	Higher proportion of predicted prednisolone-resistant cells at diagnosis associates with increased relapse risk	(38)
	Drug resistance gene signature (including *G0S2*, *ATPV06A* and *ITK*)	KRAS signaling, heme metabolism and lineage-inappropriate gene expression	Diagnostic transcriptomic signature correlate with ex vivo resistance to antimetabolites, glucorticoids, and doxorubicin, and associates with relapse-free survival	(39)
Post-transcriptional regulation	miR-150, miR-99a	Cell proliferation, apoptosis, cell cycle regulation, apoptosis	Low expression associated with relapse	(43)
	miR-124-3p	Predicted to target IFI44L (interferon pathway)	Upregulated in diagnostic marrow of patients with early relapse	(30)
	miR-103a-3p, miR-486-3p	Predicted to target *HOXA7*, *S100A10* (developmental dysregulation, cell cycle progression, stress-adaptive survival)	Downregulated in diagnostic marrow of patients with early relapse	(30)
	LINC00152	Predicted to target genes involved in cell adhesion and kinase signaling	High expression predictive of early relapse	(44)
	LINC01013	Undefined	Low expression predictive of early relapse	(44)
	LINC00652 + 6 mRNAs (*INSL3, NIPAL2, REN, RIMS2, RPRM, SNAP91*)	Coordinated network regulating metabolic pathways, membrane trafficking, and stress adaptation	Integrated signature stratifies high- vs low-risk patients for early relapse and survival	(45)
	miRNA-low cluster (MLC)	MYC activation, oxidative phosphorylation upregulation, chemoresistance	Independent predictor of relapse; dynamic evolution under therapy	(46)
	surviBALL model (lnckb.8576, lnckb.32044, lnckb.61426, lnckb.66409 and lnckb.83321)	Correlates with *NOTCH3* (transcriptional regulator), *CFD* (alternative complement pathway, immune and metabolic modulation), *PVR* (cell adhesion and immune regulation)	Combined expression model stratifies 3 risk groups, high risk shows poorer EFS	(47)
	Non-coding RNAs panel (hsa-miR-27a-5p, hsa-mir-142, and hsa-miR-411-5p, hsa_circ_0022620, hsa_circ_0061990, and hsa_circ_0039036)	Undefined	Independent predictor of relapse, its combination with clinical features formed a superior risk stratification model	(49)

## 2 Diagnostic transcriptomic signatures associated with relapse

### 2.1 Cell death dysregulation and stress-adaptive survival mechanisms

Evasion of programmed cell death (PCD) represents a central mechanism underlying treatment failure and relapse in pediatric BCP-ALL. While conventional chemotherapy relies heavily on the induction of apoptosis, accumulating evidence indicates that relapse is associated with coordinated dysregulation across multiple PCD modalities, collectively influencing leukemic cell survival, stress tolerance, and therapeutic resistance.

Large-scale transcriptomic analyses of diagnostic BCP-ALL bone marrow from Therapeutically Applicable Research to Generate Effective Treatments (TARGET), Gene Expression Omnibus (GEO), the Genotype-Tissue Expression (GTEx), and authors’ local databases, integrating bulk RNA-sequencing and single-cell RNA-sequencing, have identified relapse-associated alterations across 16 distinct PCD patterns that are associated with subsequent relapses, with nine showing strong prognostic relevance (12). Interestingly, apoptosis-related transcriptomic signatures have been associated with increased relapse risk, even though classical models would predict that impaired apoptosis favors leukemic survival. This apparent contradiction may reflect that relapse-prone leukemic cells exhibit dysregulated, rather than uniformly suppressed apoptotic signaling, enabling them to evade therapy while maintaining adaptive plasticity (12). Supporting this, prior studies have linked alterations in the Bax/Bcl-2 ratio to remission failure and relapse in ALL (13, 14), highlighting that both pro- and anti-apoptotic components may contribute to relapse-prone states depending on the cellular and treatment context.

Beyond apoptosis, autophagy is another key programmed cell death pathway that plays a central but context-dependent role in B-ALL. It can function either as a tumor-suppressive mechanism by removing damaged cellular components or as a survival pathway that promotes leukemic persistence and therapy resistance. Programmed cell death transcriptomic signatures suggest that autophagy is negatively associated with relapse risk, indicating a protective role in B-ALL (12), potentially facilitating effective elimination of leukemic cells under therapeutic stress. While glucocorticoids can induce autophagy-mediated cell death as part of BCP-ALL induction therapy (15), dysregulated or adaptive autophagy has been shown to promote cell proliferation and drug resistance e.g. towards L-asparaginase in *ETV6::RUNX1* subtype, further complicating treatment outcomes (16). These findings highlight that the functional role of autophagy in leukemia is context-dependent.

Several other programmed cell death (PCD) pathways—including ferroptosis, necroptosis, entotic cell death, and alkaliptosis—were identified as protective factors in transcriptomic-based analyses of BCP-ALL, with intact signaling limiting leukemic cell survival, whereas neurotic cell death, oxeiptosis, and NETosis are associated with higher relapse risk (12). Ferroptosis has also been highlighted in another study involving Philadelphia chromosome–negative BCP-ALL, with transcriptomic analyses of ferroptosis-related genes revealing heterogeneity with potential prognostic relevance (17).

These contrasting associations may reflect differences in the immunological and microenvironmental consequences of distinct PCD modalities. Certain forms of PCD, including ferroptosis and necroptosis, can be immunogenic, promoting the release of damage-associated molecular patterns, which enhance immune recognition and clearance of tumor cells (18). In contrast, other death programs, such as oxeiptosis and NETosis, may favor tolerogenic clearance or chronic inflammatory remodeling, creating a niche that supports the survival of residual leukemic cell (19). Collectively, these findings highlight that relapse is not solely due to impaired apoptosis or autophagy, but rather the selective rewiring of multiple programmed-cell death programs that favor leukemic survival under therapeutic stress. Notably, a transcriptome-based cell death index (CDI) score derived from seven PCD-related genes (*BIK*, *TSPO*, *BCL2L2*, *PIP4K2C*, *MLKL*, *STAT2*, and *WWOX*) independently predicts relapse risk, outperforming several conventional clinical variables, suggesting that early transcriptional states governing cell death susceptibility may serve as robust biomarkers for relapse prediction at diagnosis (12).

Complementing transcriptomic analyses of PCD programs, Weng et al. (20) reported that expression of the tumor suppressor *TP53*, a classical regulator of apoptosis, cell cycle arrest, and DNA repair, also predicts relapse risk in childhood ALL. In their cohort of 114 BCP-ALL and 32 T-ALL patients, high *TP53* expression at diagnosis was associated with inferior complete remission rates, overall survival, and relapse-free survival, independent of conventional clinical risk factors. However, it should be noted that the researchers did not assess *TP53* mutation status, and the targeted qPCR assay, which focused on the DNA-binding domain, cannot distinguish functionally distinct splice variants. Some variants, such as Δ40p53, which have been observed to be elevated in relapsed ALL (21), might act as dominant-negative inhibitors of tumor suppressive activity. Thus, while high *TP53* expression at diagnosis suggests a correlation to relapse risk, functional studies and more detailed mutation and isoform characterization are needed to fully interpret its prognostic significance in childhood ALL.

Together, dysregulation of intrinsic PCD pathways and stress-adaptive survival mechanisms, reflected in early transcriptomic signatures, may promote leukemic persistence under therapeutic stress, contributing to treatment resistance and increased relapse risk.

### 2.2 Immune modulation and metabolic adaptation

Increasing evidence suggests that relapse in BCP-ALL is shaped by both extrinsic bone marrow microenvironment remodeling and leukemia-intrinsic immune-evasion programs, often intertwined with metabolic adaptation. While bulk transcriptomic analyses cannot fully distinguish leukemia-intrinsic effects from microenvironment changes, they provide valuable insights into early immune and metabolic states associated with relapse.

Transcriptomic deconvolution of diagnostic and relapsed ALL bone marrow samples using CIBERSORT revealed significant shifts in immune cell composition, most notably a reduction in naive B cells and an expansion of neutrophils at relapse (22). Given the high blast burden in bulk RNA-seq samples, the reported reduction in naive B cells may reflect true depletion of normal B cells or misestimation by transcriptomic deconvolution, since leukemic blasts share gene expression features with naive B cells; therefore, these findings should be interpreted cautiously. Additional microenvironment changes associated with higher relapse risk, as reported by Luo et al. (12), include increased plasma cells and monocytes, reduced M2 macrophages, eosinophils, and neutrophils, and significant differences in stromal cell signatures. Together, these findings suggest a progressive disruption of normal immune homeostasis and potential remodeling of both innate and adaptive compartments. These immune alterations were linked to metabolic regulators, with *SIRT3* and *NADSYN1*, both involved in NAD^+^ metabolism and mitochondrial function, showing negative correlations with naïve B-cell abundance, whereas *PARP6* expression correlated positively with neutrophil infiltration (22). These observations underscore a close relationship between metabolic state and immune cell composition in the context of B-ALL relapse.

Complementing these microenvironmental observations, leukemia-intrinsic transcriptional programs also contribute to immune modulation and relapse risk. The high-risk diagnostic transcriptional states, as captured by the transcriptome-based programmed cell death index (CDI) by Luo and colleagues, correlate with altered bone marrow immune infiltration and stromal interactions (12). At the molecular level, these high-risk transcriptional states correlated positively with metabolic and proliferative pathways, including glycolysis, fatty acid metabolism, oxidative phosphorylation, MTORC1, and MYC signaling, while showing inverse correlations with interferon and inflammatory pathways (12). Together, these findings suggest that relapse-prone leukemias may adopt metabolically active yet immunologically constrained states, in which dysregulated PCD signaling, suppressed interferon responses, and immune-evasive programs collectively support leukemic persistence.

These observations are further supported by TIMD4, a cell-surface glycoprotein involved in immune tolerance and apoptotic cell clearance. High *TIMD4* expression at diagnosis was independently predictive of early relapse among 87 children with low-risk *ETV6::RUNX1*-positive B-cell ALL (23). Although bulk transcriptomic profiling cannot definitively resolve whether *TIMD4* expression originates from leukemic blasts or tumor-associated myeloid cells, its known roles in attenuating antigen presentation and promoting immune tolerance are directionally consistent with the suppressed interferon signaling and altered immune infiltration patterns observed in high-relapse risk transcriptional states described above. Moreover, TIMD4 has been linked in other malignancies to activation of metabolic pathways such as oxidative phosphorylation and Wnt/β-catenin signaling (24, 25), aligning with the metabolically active yet immunologically constrained phenotype characteristic of relapse-prone leukemias.

Beyond relapse risk alone, immune-associated transcriptomic states at diagnosis have also been linked to variability in chemotherapy response in pediatric BCP-ALL. Jing and Li (26) reported that in a large integrative analysis of diagnostic marrow from 456 samples across five independent cohorts, a 59-gene expression signature derived from relapse-free survival modelling stratified patients into high- and low-risk groups with significantly different relapse-free survival outcomes. The signature included genes involved in chemokine and cytokine signaling (*SOCS3*), cell-cycle regulation (*CDK6*), intracellular trafficking (*RAB7A, RAB27B*), and lineage-associated markers (*MME/CD10*), underscoring the contribution of immune regulation and cellular stress responses to relapse biology.

Extending these observations, transcriptomic profiling of diagnostic bone marrow has revealed that CNS relapse may also be shaped by early immune and antigen-presentation programs. Analysis of TARGET RNA-seq data comparing ALL patients who later developed isolated CNS relapse with those who did not, identified differential expression of genes enriched in antigen processing and presentation, MHC class II receptor activity, and positive regulation of T-cell aggregation (27). Multivariate modelling using identified hub genes identified *CD19*, *PPARG*, and *GNG12* as independent predictors of CNS relapse (27). PPARG, a key regulator of lipid metabolism and mitochondrial function with established anti-proliferative and pro-apoptotic roles, was significantly downregulated in CNS-relapsed cases and accompanied by activation of PI3K–Akt signaling, suggesting metabolic rewiring that may favor leukemic survival. In parallel, GNG12, a regulator of heterotrimeric G-protein signaling involved in endothelial junction integrity, was upregulated, implicating altered leukemic–endothelial interactions and potential facilitation of blood–brain barrier crossing. While CD19 is a canonical B-cell lineage marker and therapeutic target, its elevated expression at diagnosis was paradoxically associated with increased CNS relapse risk. This observation suggests that beyond lineage identification, heightened *CD19* expression may reflect altered B-cell receptor signaling, immune interaction dynamics, or selection of leukemic subpopulations with enhanced CNS tropism. These findings are consistent with clinical observations that CD19-directed therapies are effective in CNS disease (28), underscoring the biological relevance of CD19-associated immune programs in CNS relapse.

Collectively, these findings support a proposed model in which extrinsic microenvironmental remodeling and intrinsic leukemia programs - including early immune tolerance and alterations in antigen presentation programs, cooperate with metabolic adaptations to facilitate leukemic persistence, influence treatment responsiveness, and drive subsequent relapse.

### 2.3 Developmental dysregulation and stemness

Beyond survival and immune escape, relapse in pediatric BCP-ALL is strongly linked to dysregulation of developmental and differentiation programs, which can favor persistence of immature, therapy-tolerant leukemic blasts.

Early transcriptome-wide analyses in the COG 9906 ALL cohort demonstrated that developmental and stemness-associated transcriptional programs contribute to relapse risk in pediatric B-precursor ALL. A 38-gene expression classifier predictive of relapse-free survival, when combined with end-induction minimal residual disease (MRD) measurements, stratified high-risk patients at diagnosis, highlighting dysregulated B-cell differentiation, progenitor-like signaling, and stemness-associated states (e.g., *BMPR1B*, *CTGF/CCN2*, *IGJ*, *TSPAN7*) independently of classical cytogenetic abnormalities (29). Patients with worse outcome exhibited elevated expression of genes involved in adaptive cell signaling response to transforming growth factor 𝛽, stem cell function, B-cell development and differentiation, whereas low-risk patients showed relatively higher expression of tumor suppressor and signaling regulatory genes (*NR4A3*, *BTG3*, *RGS1*, *RGS2*). Notably, all 20 cases harboring the t(1,19)(*TCF3::PBX1*) translocation were classified as low-risk by the combined gene signature and MRD, yet 8 of these patients experienced relapse, illustrating that residual relapse risk persists in specific developmental subtypes despite transcriptomic and MRD-based risk stratification.

Building on these observations, more recent multi-cohort transcriptomic analyses have further delineated developmental and stemness-associated leukemic progenitor states contributing to relapse. A study by Huang et al. (30) using TARGET and GEO databases identified aberrant activation of homeobox genes, altered cell-cycle regulators, and stress-adaptive pathways in diagnostic blasts, leading to a four-gene risk model (*HOXA7*, *S100A10*, *S100A11*, *IFI44L*) that predicts early relapse independent of minimal residual disease. HOXA7, a homeobox transcription factor essential for hematopoietic development, was positively associated with early relapse risk and has been implicated in differentiation blockade and poor outcomes in a mixture of leukemias, particularly in *MLL*-rearranged (*KMT2A*-r) leukemias (30, 31). In parallel, *S100A10* and *S100A11* were also upregulated in patients with early relapse. These calcium-binding proteins are involved in cytoskeletal dynamics, cell cycle progression, and stress adaptation, with elevated expression associated with chemoresistance across various solid cancers and leukemias (30, 32). Conversely, *IFI44L*, an interferon-stimulated gene, was downregulated in patients with early relapse, suggesting a convergence of developmental dysregulation with altered immune signaling in relapse-prone populations.

Together, these findings indicate that transcriptional states reflecting immature, proliferative, and stress-adaptive programs in diagnostic marrow may also serve as early predictors of relapse. In line with these observations, a study analyzing 35 matched diagnosis-relapse marrow samples - primarily BCP-ALL with three T-ALL cases- found that early relapse clones maintained transcriptional similarity to their diagnostic sample, particularly in cell-cycle and proliferation pathways, whereas late-relapse clones diverged more extensively (33). These findings suggest that early-relapse risk may be associated with pre-existing transcriptional programs detected at diagnosis, reinforcing the importance of developmental and proliferative states in relapse risk. Echoing this, single-cell transcriptomic analyses reveal that in *KMT2A*-rearranged infant BCP-ALL, most leukemic blasts at diagnosis exhibit transcriptional similarity to early lymphocyte precursor (ELP) cells (34). This primitive developmental state is maintained in resistant disease and at relapse, highlighting that a persistent immature transcriptional program at the single-cell level underlies aggressive disease biology and may contribute to therapy tolerance and relapse risk.

### 2.4 Drug resistance

Drug resistance represents the fundamental biological barrier to successful treatment of relapsed childhood ALL, with multiple molecular mechanisms contributing to therapeutic failure. Transcriptomic analyses of diagnostic bone marrow reveal that early transcriptomic states can anticipate drug resistance. For instance, in two independent cohorts of pediatric BCP-ALL, within the 59-gene expression signature derived from relapse-free survival modeling, coordinated expression of four genes in diagnostic marrow, namely *B4GALT5*, *CDK6*, *PDZD8*, and *RAB7A*, was associated with prednisolone response (26). The B4GALT family, in particular, has been shown previously to mediate multidrug resistance among myeloid leukemia cells by regulating the Hedgehog pathway (35), which can be therapeutically targeted using a hedgehog antagonist such as cyclopamine (36).

In parallel, in a study by Paugh et al. (37), transcriptomic analyses of diagnostic BCP-ALL samples revealed significantly higher expression of *CASP1* and its activator *NLRP3* in glucocorticoid-resistant leukemia cells, driven by promoter hypomethylation. Overexpression of *CASP1* leads to cleavage of the glucocorticoid receptor, diminishing glucocorticoid-induced transcriptional responses and conferring resistance (37). These findings highlight a transcriptional mechanism of drug resistance at diagnosis that may inform early risk stratification.

Single-cell transcriptomic analyses provide additional resolution by revealing preexisting therapy-resistant subpopulations within diagnostic samples. In *MLL*/*KMT2A*-rearranged infant BCP-ALL, Candelli et al. (2022) (32) used single-cell RNA sequencing of nearly 600 diagnostic bone marrow and peripheral blood samples to classify leukemic cells along a prednisone-sensitive to -resistant continuum (38). Cells predicted to be resistant exhibited quiescent, stemness-associated transcriptional programs and basal glucocorticoid response signatures, and a higher proportion of these cells at diagnosis correlated with subsequent relapse. These findings highlight how diagnostic transcriptional heterogeneity at single-cell resolution can uncover therapy-resistant subpopulations that may be obscured in bulk analyses.

Further integrative analyses combining ex vivo drug response profiling with transcriptomic and epigenomic signatures in diagnostic BCP-ALL samples from 597 Swedish patients demonstrated that intrinsic molecular states at diagnosis predict resistance to antimetabolites (e.g., cytarabine thioguanine), glucocorticoid (e.g., prednisolone, dexamethasone), and doxorubicin (39). Resistance to these drugs was independently associated with reduced relapse-free survival. Diagnostic leukemic marrow associated with later drug resistance showed aberrant expression patterns linked to KRAS activation (e.g., *GS02*), heme metabolism (e.g., *ATPV06A*), and atypical expression of T-cell-related gene (e.g., *ITK*), reflecting complex transcriptional reprogramming underlying drug resistance (39).

Collectively, these observations suggest that diagnostic transcriptomic and epigenomic profiling can provide early insights into drug resistance, revealing cell-intrinsic sensitivity and molecular pathways that may be targeted to overcome treatment failure. While classical resistance mutations (e.g., *NTC52* nucleotidase enzyme, *NR3C1* or *NR3C2* glucocorticoid receptors, *FPGS* involved in folate metabolism) typically emerge during relapse of B- and T-ALL (40–42), these studies underline the predictive value of diagnostic transcriptomic states, particularly for glucocorticoid and other conventional therapies. Nevertheless, the heterogeneity of resistance mechanisms across drug classes and patients may limit the predictive power of any single transcriptomic signature, underscoring the need for personalized approaches that integrate transcriptomic profiling with functional drug response assays and longitudinal monitoring.

### 2.5 Post-transcriptional regulation

In addition to transcriptional dysregulation, post-transcriptional mechanisms play a critical role in shaping relapse-prone leukemic states. Through miRNAs and other non-coding RNAs, leukemic cells can fine-tune the expression of key relapse-associated genes identified in diagnostic marrow, reinforcing transcriptional programs that confer survival, developmental immaturity, stress adaptation, metabolic fitness, immune evasion, and treatment resistance. A systematic review of miRNA deregulation in childhood ALL highlighted recurrent patterns associated with relapse and prognosis. Specifically, downregulation of miR-150 and miR-99a at diagnosis was correlated with higher relapse risk, whereas upregulation of miR-708 was associated with lower relapse risk, supporting the potential of miRNA signatures measured at diagnosis to refine risk stratification in BCP-ALL (43).

Analysis of miRNA-sequencing data from diagnostic B-ALL samples in the TARGET dataset further revealed that miR-124-3p was upregulated in patients who later experienced early relapse and predicted to target *IFI44L*, an interferon-stimulated gene downregulated in early relapse (see Developmental dysregulation and stemness domain) (30). Conversely, miR-103a-3p and miR-486-3p were downregulated in diagnostic marrow of early relapse patients, corresponding to derepression of *HOXA7* and *S100A10* (30), which are implicated in developmental dysregulation, cell cycle progression, and stress-adaptive survival. These findings illustrate how discrete miRNA–mRNA interactions can fine-tune transcriptional programs that predispose leukemias to early relapse.

Beyond discrete miRNA interactions, long non-coding RNAs (lncRNAs) may also modulate post-transcriptional networks in the BCP-ALL relapse predisposition context. For example, high expression of LINC00152 and low expression of LINC01013 were identified in a microarray-based study as associated with early relapse and early mortality (44). LncRNA-mRNA co-expression analysis suggested that LINC00152 may regulate genes involved in cell substrate adhesion and peptidyl-tyrosine autophosphorylation, linking post-transcriptional regulation to cellular processes relevant to leukemic persistence, whereas the functional role of LINC01013 remains unclear.

Beyond individual lncRNAs, a lncRNA–mRNA–based prognostic classifier was developed in a multi-cohort transcriptomic analysis of B-ALL and T-ALL, integrating TARGET and GEO datasets, using diagnostic bone marrow samples to predict recurrence and survival outcomes (45). A seven-gene signature comprising one lncRNA (LINC00652) and six protein-coding genes (*INSL3*, *NIPAL2*, *REN*, *RIMS2*, *RPRM*, and *SNAP91*) robustly stratified patients into high- and low-risk groups for recurrence and overall survival, independent of conventional clinical variables. Network-based analyses suggested that LINC00652 functions as a regulatory hub coordinating coexpression of relapse-associated genes, with enrichment in pathways linked to metabolic regulation, membrane trafficking, and stress-related cellular regulation. Although the precise cellular mechanisms remain incompletely defined, these findings indicate that noncoding RNA–mediated transcriptional coordination may stabilize relapse-prone states characterized by altered metabolic and stress-adaptive programs already present at diagnosis, reinforcing the value of integrated non-coding RNA signatures for early relapse prediction.

On a larger scale, integrated global miRNA- and mRNA-sequencing analyses identified a distinct “miRNA-low cluster” (MLC) in diagnostic B-ALL blasts, characterized by global downregulation of miRNAs and independently associated with inferior event-free survival, independent of existing subtypes, driver mutations, and methylation alterations, including within the otherwise favorable hyperdiploid ALL (46). Notably, a subset of patients classified as non-MLC at diagnosis transitioned to an MLC profile at relapse, underscoring that this miRNA-low state is not only predictive when present at diagnosis but can also be progressively acquired under treatment pressure, reinforcing its biological relevance to relapse evolution. Integrated transcriptomic analysis further revealed that the MLC was characterized by enrichment of MYC target and oxidative phosphorylation genes, linking post-transcriptional dysregulation to proliferative and metabolic reprogramming observed in relapse-prone leukemias. Mechanistically, reduced expression of the miRNA-processing enzyme DICER1 may underlie this phenotype, and accompanying reductions in intron retention could contribute to aberrant mRNA processing, collectively promoting chemoresistance and survival.

Extending observations of a global high-risk non-coding RNA landscape, the surviBALL model demonstrates that a lncRNA-only signature measured at diagnosis can capture relapse risk with high prognostic accuracy (47). The model comprises five lncRNAs whose combined expression predicts 5-year event-free survival across multiple independent pediatric B-ALL cohorts, treated with different protocols (SEHOP-PETHEMA, DFCI, and COG group protocols), independently of molecular subtype and MRD status. Although the biological functions of these lncRNAs remain largely uncharacterized, guilt-by-association analyses provide insight into their potential pathways. Notably, lnckb.61426 correlates with *NOTCH3*, encoding a transcriptional regulator previously reported to be overexpressed in high-risk ALL (48), while lnckb.32044 and lnckb.66409 correlates with complement factor D (*CFD*), encoding a serine protease linking innate immune activation with metabolic and inflammatory signaling. In addition, lnckb.66409 correlates with *PVR* (*CD155*), encoding a transmembrane glycoprotein involved in cell adhesion and immune checkpoint interactions, linking lncRNA dysregulation to immune evasion mechanisms that may facilitate leukemic persistence (47).

Complementing marrow-based signatures, a recent plasma-based non-coding RNA panel offers a non-invasive approach for early relapse prediction. Using diagnostic plasma from pediatric ALL patients, three miRNAs (hsa-miR-27a-5p, hsa-mir-142, and hsa-miR-411-5p) and three circRNAs (hsa_circ_0022620, hsa_circ_0061990, and hsa_circ_0039036) were identified as predictive of relapse, with expression increasing from healthy controls to newly diagnosed to relapsed patients (49). Validation in independent cohorts confirmed that the panel combining all six non-coding RNAs outperformed individual markers, and integration with clinical risk features further enhanced predictive accuracy, demonstrating the potential for early, non-invasive risk stratification at diagnosis.

Together, these study findings highlight that post-transcriptional dysregulation is also a key feature of relapse risk in ALL. Beyond changes in expression of protein-coding genes, non-coding RNAs, including miRNAs and lncRNAs, may also act as mechanistic drivers of relapse-associated signatures, impacting cell death and survival, developmental/differentiation programs, metabolic adaptation, immune regulation, and drug resistance. These coordinated transcriptomic alterations likely reflect system-level rewiring of interconnected pathways rather than isolated genetic lesions or pathway disruption, highlighting the value of post-transcriptional biomarkers as complementary tools for refining early risk stratification and relapse in ALL.

## 3 Clonal evolution and subclone dynamics

While diagnostic transcriptomic biomarkers or signatures can provide valuable prognostic information for relapse risk, it is important to acknowledge that relapsed childhood ALL frequently follows complex clonal evolutionary patterns, diverging from linear progression models. Analysis of matched diagnosis-relapse samples demonstrates that concordance between the post-chemotherapy relapse leukemia clone and the primary diagnosis clone occurs only in 8% of patients, with the majority of relapse clones evolving from minor clones at diagnosis or from pre-leukemic ancestral clones (50). In a landmark study involving children with B-ALL (n=67) and T-ALL (n=25), relapse-driving clone originating from a minor diagnostic subclone was reported in 50% of cases, from a major clone in 27%, and was multiclonal in 18% (51), illustrating the heterogeneous origins of relapse. Consequently, a proportion of relapses may not be fully predicted by baseline expression profiles alone.

These minor subclones at diagnosis, termed diagnosis relapse-initiating (dRI) clones, exhibit distinct biological properties, including altered metabolic states, stemness signature, and drug resistance, which may overlap with features captured by high-risk diagnostic transcriptomic signatures (52, 53). These findings suggest that diagnostic transcriptomic biomarkers not only reflect intrinsic relapse risk but may also partially anticipate the cellular adaptations that enable survival under therapeutic pressure. However, the heterogeneity and dynamic evolution of these clones imply that transcriptomic snapshots at diagnosis have inherent limitations in fully capturing the relapse potential. While bulk transcriptomic analyses can reveal global dysregulated programs, emerging single-cell RNA sequencing approaches provide resolution to dissect cellular heterogeneity at diagnosis, enabling identification of rare dRI clones and their unique transcriptomic reprogramming.

In short, these complementary approaches support an integrative framework in which bulk transcriptomic signatures define relapse-associated programs, single-cell analyses resolve rare relapse-initiating subclones, and longitudinal monitoring through MRD measurements and clonal tracking provides functional evidence of treatment response and clonal persistence. Integrating these layers of information may improve stratification and provide a more comprehensive understanding of relapse biology, supporting more precise prediction of relapse risk in pediatric BCP-ALL.

## 4 Conclusion

Across the biological domains highlighted in this review, a recurring observation is that relapse risk is most accurately captured by composite transcriptional signatures rather than individual genes. Although the constituent genes of these models differ—reflecting programmed cell death, immune regulation, developmental arrest, metabolic adaptation, or drug resistance —they converge on shared biological themes that promote leukemic persistence under therapeutic stress. Such multi-gene risk scores likely function as integrative readouts of coordinated pathway dysregulation present at diagnosis, reinforcing the concept that relapse is encoded at a systems level rather than driven by isolated molecular events.

However, these findings should be interpreted with caution. Most analyses were retrospective, performed on single or limited datasets, often with small sample sizes and incomplete clinical annotations. Included cohorts differ in patient age distribution, treatment regimens, transcriptomic platforms (RNA-seq versus microarray), and analytical pipelines, which may influence the comparability of individual gene-level findings. Nevertheless, the overall age distribution is broadly similar, and contemporary childhood BCP-ALL treatment generally follows a shared multi-phase chemotherapy backbone, providing a common biological context for interpreting relapse-associated transcriptomic signatures. Despite platform- or cohort-specific differences in gene-level results, the reported signatures consistently point toward similar biological domains, supporting the overarching patterns highlighted in this review. Many studies relied on bulk transcriptomic data, leaving uncertainty about the specific cell populations driving the observed changes. Functional validation of gene signatures, candidate gene markers, or non-coding RNAs is largely lacking, and mechanistic links to biological pathway changes remain to be experimentally confirmed. Future combined efforts should focus on prospective validation in larger, independent cohorts, incorporation of single-cell transcriptomic approaches, and experimental studies to clarify causal mechanisms and assess the predictive utility of these molecular signatures in clinical practice. Building on these efforts, systematic analyses across multiple biological domains highlighted in this review across large datasets could help identify the most prominent or actionable pathways, evaluate potential interactions, and determine their translational utility when integrated with MRD measurements and existing clinical risk models. Should such an endeavor prove to be successful, it would herald a significant leap in precision therapy for patients with ALL, and perhaps, for other cancers as well.

## References

[1] Schwab C Harrison CJ . Advances in B-cell precursor acute lymphoblastic leukemia genomics. Hemasphere. (2018) 2:e53. doi: 10.1097/hs9.0000000000000069. PMID: 31723781 PMC6746003

[2] Ding F Deng L Xiong J Cheng Z Xu J . Analysis of global trends in acute lymphoblastic leukemia in children aged 0–5 years from 1990 to 2021. Front Pediatr. (2025) 13:1542649. doi: 10.3389/fped.2025.1542649. PMID: 40181994 PMC11966407

[3] Yeoh AEJ Lu Y Chin WHN Chiew EKH Lim EH Li Z . Intensifying treatment of childhood B-lymphoblastic leukemia with IKZF1 deletion reduces relapse and improves overall survival: Results of Malaysia-Singapore ALL 2010 study. J Clin Oncol. (2018) 36:2726–35. doi: 10.1200/jco.2018.78.3050. PMID: 30044693

[4] Vrooman LM Silverman LB . Treatment of childhood acute lymphoblastic leukemia: Prognostic factors and clinical advances. Curr Hematol Malig Rep. (2016) 11:385–94. doi: 10.1007/s11899-016-0337-y. PMID: 27502091

[5] Enshaei A O'Connor D Bartram J Hancock J Harrison CJ Hough R . A validated novel continuous prognostic index to deliver stratified medicine in pediatric acute lymphoblastic leukemia. Blood. (2020) 135:1438–46. doi: 10.1182/blood.2019003191. PMID: 32315382

[6] Vora A Goulden N Mitchell C Hancock J Hough R Rowntree C . Augmented post-remission therapy for a minimal residual disease-defined high-risk subgroup of children and young people with clinical standard-risk and intermediate-risk acute lymphoblastic leukaemia (UKALL 2003): a randomised controlled trial. Lancet Oncol. (2014) 15:809–18. doi: 10.1016/s1470-2045(14)70243-8. PMID: 24924991

[7] Jeha S Pei D Choi J Cheng C Sandlund JT Coustan-Smith E . Improved CNS control of childhood acute lymphoblastic leukemia without cranial irradiation: St Jude Total Therapy Study 16. J Clin Oncol. (2019) 37:3377–91. doi: 10.1200/jco.19.01692. PMID: 31657981 PMC7351342

[8] Pieters R de Groot-Kruseman H Van der Velden V Fiocco M van den Berg H de Bont E . Successful therapy reduction and intensification for childhood acute lymphoblastic leukemia based on minimal residual disease monitoring: Study ALL10 from the Dutch Childhood Oncology Group. J Clin Oncol. (2016) 34:2591–601. doi: 10.1200/jco.2015.64.6364. PMID: 27269950

[9] Yeoh AE Ariffin H Chai EL Kwok CS Chan YH Ponnudurai K . Minimal residual disease-guided treatment deintensification for children with acute lymphoblastic leukemia: Results from the Malaysia-Singapore acute lymphoblastic leukemia 2003 study. J Clin Oncol. (2012) 30:2384–92. doi: 10.1200/jco.2011.40.5936. PMID: 22614971

[10] Ariffin H Chiew EKH Oh BLZ Lee SHR Lim EH Kham SKY . Anthracycline-free protocol for favorable-risk childhood ALL: A noninferiority comparison between Malaysia-Singapore ALL 2003 and ALL 2010 studies. J Clin Oncol. (2023) 41:3642–51. doi: 10.1200/jco.22.02347. PMID: 37276496

[11] Maloney KW Devidas M Wang C Mattano LA Friedmann AM Buckley P . Outcome in children with standard-risk B-cell acute lymphoblastic leukemia: Results of Children's Oncology Group Trial AALL0331. J Clin Oncol. (2020) 38:602–12. doi: 10.1200/jco.19.01086. PMID: 31825704 PMC7030893

[12] Luo Y Tan L Meng C Gao J Chen H Fang R . Integrating bulk RNA-seq and scRNA-seq data to explore diverse cell death patterns and develop a programmed cell death-related relapse prediction model in pediatric B-ALL. Sci Rep. (2025) 15:5620. doi: 10.1038/s41598-025-86148-y. PMID: 39955305 PMC11829959

[13] Stamati L Avgeris M Kosmidis H Baka M Anastasiou T Piatopoulou D . Overexpression of BCL2 and BAX following BFM induction therapy predicts ch-ALL patients' poor response to treatment and short-term relapse. J Cancer Res Clin Oncol. (2015) 141:2023–36. doi: 10.1007/s00432-015-1982-6. PMID: 25982455 PMC11824082

[14] Prokop A Wieder T Sturm I Essmann F Seeger K Wuchter C . Relapse in childhood acute lymphoblastic leukemia is associated with a decrease of the Bax/Bcl-2 ratio and loss of spontaneous caspase-3 processing *in vivo*. Leukemia. (2000) 14:1606–13. doi: 10.1038/sj.leu.2401866. PMID: 10995007

[15] Laane E Tamm KP Buentke E Ito K Kharaziha P Oscarsson J . Cell death induced by dexamethasone in lymphoid leukemia is mediated through initiation of autophagy. Cell Death Differ. (2009) 16:1018–29. doi: 10.1038/cdd.2009.46. PMID: 19390558

[16] Polak R Bierings MB van der Leije CS Sanders MA Roovers O Marchante JRM . Autophagy inhibition as a potential future targeted therapy for ETV6-RUNX1-driven B-cell precursor acute lymphoblastic leukemia. Haematologica. (2019) 104:738–48. doi: 10.3324/haematol.2018.193631. PMID: 30381299 PMC6442983

[17] Hong Y Zhang L Tian X Xiang X Yu Y Zeng Z . Identification of immune subtypes of Ph-neg B-ALL with ferroptosis related genes and the potential implementation of Sorafenib. BMC Cancer. (2021) 21:1331. doi: 10.1186/s12885-021-09076-w. PMID: 34906116 PMC8670244

[18] Gao W Wang X Zhou Y Wang X Yu Y . Autophagy, ferroptosis, pyroptosis, and necroptosis in tumor immunotherapy. Signal Transduct Target Ther. (2022) 7:196. doi: 10.1038/s41392-022-01046-3. PMID: 35725836 PMC9208265

[19] Demkow U . Neutrophil extracellular traps (NETs) in cancer invasion, evasion and metastasis. Cancers (Basel). (2021) 13. doi: 10.3390/cancers13174495. PMID: 34503307 PMC8431228

[20] Weng W Zhang P Ruan J Zhang Y Ba D Tang Y . Prognostic significance of the tumor suppressor protein p53 gene in childhood acute lymphoblastic leukemia. Oncol Lett. (2020) 19:549–56. doi: 10.3892/ol.2019.11064. PMID: 31897170 PMC6924105

[21] Oh L Hainaut P Blanchet S Ariffin H . Expression of p53 N-terminal isoforms in B-cell precursor acute lymphoblastic leukemia and its correlation with clinicopathological profiles. BMC Cancer. (2020) 20:110. doi: 10.1186/s12885-020-6599-8. PMID: 32041553 PMC7011217

[22] Lin C Xu JQ Zhong GC Chen H Xue HM Yang M . Integrating RNA-seq and scRNA-seq to explore the biological significance of NAD + metabolism-related genes in the initial diagnosis and relapse of childhood B-cell acute lymphoblastic leukemia. Front Immunol. (2022) 13:1043111. doi: 10.3389/fimmu.2022.1043111. PMID: 36439178 PMC9691973

[23] Gong X Hu T Shen Q Zhang L Zhang W Liu X . Gene expression prognostic of early relapse risk in low-risk B-cell acute lymphoblastic leukaemia in children. EJHaem. (2024) 5:333–45. doi: 10.1002/jha2.872. PMID: 38633121 PMC11020147

[24] Li Y Zhang PY Yang ZW Ma F Li FX . TIMD4 exhibits regulatory capability on the proliferation and apoptosis of diffuse large B-cell lymphoma cells via the Wnt/beta-catenin pathway. J Gene Med. (2020) 22:e3186. doi: 10.1002/jgm.3186. PMID: 32187802

[25] Wang Y Wang Y Liu W Ding L Zhang X Wang B . TIM-4 orchestrates mitochondrial homeostasis to promote lung cancer progression via ANXA2/PI3K/AKT/OPA1 axis. Cell Death Dis. (2023) 14:141. doi: 10.1038/s41419-023-05678-3. PMID: 36806050 PMC9941510

[26] Jing W Li J . Identification of biomarkers for the prediction of relapse-free survival in pediatric B-precursor acute lymphoblastic leukemia. Oncol Rep. (2019) 41:659–67. doi: 10.3892/or.2018.6846. PMID: 30542734

[27] Zhang S Tu Y Lai H Chen H Tu H Li J . PPARG, GNG12, and CD19 are potential independent predictors of central nerve recurrence in childhood acute lymphoblastic leukemia. Hematology. (2023) 28:2182169. doi: 10.21203/rs.3.rs-963371/v2 36861936

[28] Qi Y Zhao M Hu Y Wang Y Li P Cao J . Efficacy and safety of CD19-specific CAR T cell-based therapy in B-cell acute lymphoblastic leukemia patients with CNSL. Blood. (2022) 139:3376–86. doi: 10.1182/blood.2021013733. PMID: 35338773 PMC11022988

[29] Kang H Chen IM Wilson CS Bedrick EJ Harvey RC Atlas SR . Gene expression classifiers for relapse-free survival and minimal residual disease improve risk classification and outcome prediction in pediatric B-precursor acute lymphoblastic leukemia. Blood. (2010) 115:1394–405. doi: 10.1182/blood-2009-05-218560. PMID: 19880498 PMC2826761

[30] Huang Y Jiazheng L Yanxin C Peifang J Ling-yan W Jianda H . Identification of early recurrence factors in childhood and adolescent B-cell acute lymphoblastic leukemia based on integrated bioinformatics analysis. Front Oncol. (2020) 10. doi: 10.3389/fonc.2020.565455. PMID: 33134167 PMC7550668

[31] Ferrando AA Armstrong SA Neuberg DS Sallan SE Silverman LB Korsmeyer SJ . Gene expression signatures in MLL-rearranged T-lineage and B-precursor acute leukemias: Dominance of HOX dysregulation. Blood. (2003) 102:262–8. doi: 10.1182/blood-2002-10-3221. PMID: 12637319

[32] Saiki Y Horii A . Multiple functions of S100A10, an important cancer promoter. Pathol Int. (2019) 69:629–36. doi: 10.1111/pin.12861. PMID: 31612598

[33] Bhojwani D Kang H Moskowitz NP Min DJ Lee H Potter JW . Biologic pathways associated with relapse in childhood acute lymphoblastic leukemia: A Children's Oncology Group study. Blood. (2006) 108:711–7. doi: 10.1182/blood-2006-02-002824. PMID: 16822902 PMC1895482

[34] Khabirova E Jardine L Coorens THH Webb S Treger TD Engelbert J . Single-cell transcriptomics reveals a distinct developmental state of KMT2A-rearranged infant B-cell acute lymphoblastic leukemia. Nat Med. (2022) 28:743–51. doi: 10.1038/s41591-022-01720-7. PMID: 35288693 PMC9018413

[35] Zhou H Ma H Wei W Ji D Song X Sun J . B4GALT family mediates the multidrug resistance of human leukemia cells by regulating the hedgehog pathway and the expression of p-glycoprotein and multidrug resistance-associated protein 1. Cell Death Dis. (2013) 4:e654. doi: 10.1038/cddis.2013.186. PMID: 23744354 PMC3698553

[36] Kobune M Takimoto R Murase K Iyama S Sato T Kikuchi S . Drug resistance is dramatically restored by hedgehog inhibitors in CD34+ leukemic cells. Cancer Sci. (2009) 100:948–55. doi: 10.1111/j.1349-7006.2009.01111.x. PMID: 19245435 PMC11158794

[37] Paugh S Erik BJ Savic D Laura BR William ET Prajwal G . NALP3 inflammasome up-regulation and CASP1 cleavage of the glucocorticoid receptor causes glucocorticoid resistance in leukemia cells. Nat Genet. (2015) 47:607–614. doi: 10.1038/ng.3283. PMID: 25938942 PMC4449308

[38] Candelli T Schneider P Garrido Castro P Jones LA Bodewes E Rockx-Brouwer D . Identification and characterization of relapse-initiating cells in MLL-rearranged infant ALL by single-cell transcriptomics. Leukemia. (2022) 36:58–67. doi: 10.1038/s41375-021-01341-y. PMID: 34304246 PMC8727302

[39] Enblad AP Krali O Gezelius H Lundmark A Blom K Andersson C . Ex vivo drug responses and molecular profiles of 597 pediatric acute lymphoblastic leukemia patients. Hemasphere. (2025) 9:e70176. doi: 10.1002/hem3.70176. PMID: 40727946 PMC12301861

[40] Julia AM Jinhua W Hogan L Jun JY Smita D Jay PP . Relapse specific mutations in NT5C2 in childhood acute lymphoblastic leukemia. Nat Genet. (2013) 45:290–294. doi: 10.1038/ng.2558. PMID: 23377183 PMC3681285

[41] Xiaotu M Michael NE Yergeau D Muzny D Hampton O Michael CR . Rise and fall of subclones from diagnosis to relapse in pediatric B-acute lymphoblastic leukaemia. Nat Commun. (2015) 6. doi: 10.1038/ncomms7604. PMID: 25790293 PMC4377644

[42] Li B Brady SW Ma X Shen S Zhang Y Li Y . Therapy-induced mutations drive the genomic landscape of relapsed acute lymphoblastic leukemia. Blood. (2020) 135:41–55. doi: 10.1182/blood.2019002220. PMID: 31697823 PMC6940198

[43] Gutierrez-Camino A Garcia-Obregon S Lopez-Lopez E Astigarraga I Garcia-Orad A . miRNA deregulation in childhood acute lymphoblastic leukemia: A systematic review. Epigenomics. (2020) 12:69–80. doi: 10.2217/epi-2019-0154. PMID: 31833405

[44] Barcenas-Lopez DA Nunez-Enriquez JC Hidalgo-Miranda A Beltran-Anaya FO May-Hau DI Jimenez-Hernandez E . Transcriptome analysis identifies LINC00152 as a biomarker of early relapse and mortality in acute lymphoblastic leukemia. Genes (Basel). (2020) 11. doi: 10.3390/genes11030302, PMID: 32183133 PMC7140896

[45] Qi H Chi L Wang X Jin X Wang W Lan J . Identification of a seven-lncRNA-mRNA signature for recurrence and prognostic prediction in relapsed acute lymphoblastic leukemia based on WGCNA and LASSO analyses. Anal Cell Pathol (Amst). (2021) 2021:6692022. doi: 10.1155/2021/6692022. PMID: 34211824 PMC8208884

[46] Kubota H Ueno H Tasaka K Isobe T Saida S Kato I . RNA-seq-based miRNA signature as an independent predictor of relapse in pediatric B-cell acute lymphoblastic leukemia. Blood Adv. (2024) 8:1258–71. doi: 10.1182/bloodadvances.2023011583. PMID: 38127276 PMC10918494

[47] Illarregi U Bilbao-Aldaiturriaga N Gutierrez-Camino A Martinez de Estibariz I Arzuaga-Mendez J Camos M . surviBALL: Exploring lncRNA expression at diagnosis for 5-year EFS risk stratification in pediatric B-ALL-a proof of concept. Mol Cell Pediatr. (2025) 12:19. doi: 10.1186/s40348-025-00210-3. PMID: 41207951 PMC12597855

[48] Takam Kamga P Dal Collo G Midolo M Adamo A Delfino P Mercuri A . Inhibition of Notch signaling enhances chemosensitivity in B-cell precursor acute lymphoblastic leukemia. Cancer Res. (2019) 79:639–49. doi: 10.1158/0008-5472.can-18-1617. PMID: 30563887

[49] Wang Y Ma X Li H Zhao J Kang M Rong L . Plasma-based transcriptomic non-coding signature for predicting relapse in pediatric acute lymphoblastic leukemia. Heliyon. (2024) 10:e41102. doi: 10.1016/j.heliyon.2024.e41102. PMID: 39759366 PMC11700237

[50] Yong-feng C Jing L Ling LX Găman M Zhenhua Z . Allogeneic stem cell transplantation in the treatment of acute myeloid leukemia: An overview of obstacles and opportunities. World J Clin cases. (2023) 11:268–291. doi: 10.12998/wjcc.v11.i2.268. PMID: 36686358 PMC9850970

[51] Waanders E Gu Z Dobson SM Antic Z Crawford JC Ma X . Mutational landscape and patterns of clonal evolution in relapsed pediatric acute lymphoblastic leukemia. Blood Cancer Discov. (2020) 1:96–111. doi: 10.1158/0008-5472.bcd-19-0041. PMID: 32793890 PMC7418874

[52] Dobson SM Garcia-Prat L Vanner RJ Wintersinger J Waanders E Gu Z . Relapse-fated latent diagnosis subclones in acute B lineage leukemia are drug tolerant and possess distinct metabolic programs. Cancer Discov. (2020) 10:568–87. doi: 10.1158/2159-8290.cd-19-1059. PMID: 32086311 PMC7122013

[53] Tremblay C Saw J Feng Y Jacqueline AB Ovini A Shokoufeh A . Targeting LMO2-induced autocrine FLT3 signaling to overcome chemoresistance in early T-cell precursor acute lymphoblastic leukemia. Leukemia. (2025) 39:577–589. doi: 10.1038/s41375-024-02491-5. PMID: 39849166 PMC11879882

